# Global stability of an SAIRD epidemiological model with negative feedback

**DOI:** 10.1186/s13662-022-03712-w

**Published:** 2022-05-12

**Authors:** Roxana López-Cruz

**Affiliations:** 1 Instituto de Investigación Científica, Universidad de Lima, Surco, Lima, Perú; 2 GI CMATVIDA, UNMSM, Lima, Perú

**Keywords:** SAIRDM model, Epidemics, Negative feedback, Information index, Stability

## Abstract

In this work, we study the dynamical behavior of a modified SIR epidemiological model by introducing negative feedback and a nonpharmaceutical intervention. The first model to be defined is the Susceptible–Isolated–Infected–Recovered–Dead (SAIRD) epidemics model and then the S-A-I-R-D-Information Index (SAIRDM) model that corresponds to coupling the SAIRD model with the negative feedback. Controlling the information about nonpharmaceutical interventions is considered by the addition of a new variable that measures how the behavioral changes about isolation influence pandemics. An analytic expression of a replacement ratio that depends on the absence of the negative feedback is determined. The results obtained show that the global stability of the disease-free equilibrium is determined by the value of a certain threshold parameter called the basic reproductive number $\mathcal{R}_{0}$ and the local stability of the free disease equilibrium depends on the replacement ratios. A Hopf bifurcation is analytically verified for the delay parameter. The qualitative analysis shows that the feedback information index promotes more changes to the propagation of the disease than other parameters. Finally, the sensitivity analysis and simulations show the efficiency of the infection rate of the information index on an epidemics model with nonpharmaceutical interventions.

## Introduction

Epidemics, due to their dynamic nature, have their basic mathematical modeling that becomes more complex depending on how more factors that characterize each disease are considered. However, there are factors external to the disease that influence its incidence, and they are generated, for example, by prejudices about vaccination campaigns with prior information (negative feedback), as in the case of the negative concept in the population regarding the vaccine against the human papillomavirus (HPV) [1, 2] or the SARS-CoV-2 vaccine [3], sometimes caused by misinformation. In this work, we propose and analyze an epidemic model in ordinary differential equations including an information index function and the effect of negative feedback on the nonpharmaceutical treatment parameter (e.g., quarantine).

The Peruvian Health Office periodically launches pharmaceutical campaigns against diseases of high incidence, for example, HPV [4]. These activities are developed with the aim of reducing the incidence of these epidemics. However, there are myths [5] among the population about some of these interventions, especially when they are more effective if they are given at an early age, for prevention of sexually transmitted disease, and parents do not believe it necessary to opt for this intervention. This happens with the HPV vaccine.

One of the current concerns in Perú is the coronavirus disease 2019 (COVID-19) pandemic, which has proven the low level of response of our health system [6]. However, after 12 months, these needs have already been overcome, but we continue with the infections. The second part of the strategy against the pandemic translates into the protection of all people who have not yet been infected, and for this, it is necessary to have an efficient vaccine (pharmaceutical intervention) whose quality verification is taking time moreover with the new variants. The information that is spread about the availability of the vaccines that are now in the approval process confirms to us how difficult it will be to have them. This type of information can affect the credibility of people [2], leading them to neglect precautions with regard to cleaning and isolation (nonpharmaceutical measures or interventions), which would influence the incidence of the disease. This results in negative information (feedback). The mathematical and simulation results provide us with various scenarios of the dynamics of the disease, which will help us show the importance, for example, of good information regarding isolation.

There are some publications that analyze how negative feedback affects the incidence of diseases [7–11]. D’Onofrio et al. [9] analyzed an SIR-type model, and Vargas-De-Leon and d’Onofrio [11] treated an SEIR-type model, both with negative feedback processes. The first proposes three types of information indices, but all they analyze their models with ordinary differential equations with a linear incidence rate with respect to the information index. In this work, we use a type of nonlinear information index that gives rise to an epidemic model considering the isolation rate with negative feedback.

On the other hand, we have very difficult times in the world, with the occurrence of COVID-19, an emerging communicable disease. Unlike other diseases, COVID-19 has become a pandemic with terrible results and, consequently, has reached almost worldwide and has been severely affected by the collapse of the health system. In this case the negative feedback should be represented by the reaction of the population to the necessary strict quarantine, which would affect the incidence of the disease [12].

Buonomo [13] studied the influence of information about vaccination into a SIRI model taking into account the behavioral changes of individuals. Owolabi et al. [14] explained the adverse effects associated with antiviral treatment using a quantity termed the total control reproduction number in HIV-related cancer-immune system dynamics; also see Naik et al. [15]. Nuugulu et al. [16] analyzed a fractional SEIR model that studies the worst case characterized by ineffective COVID-19 control mechanisms satisfying the chaotic nature observed in the spread of COVID-19.

Finally, in the paper a modified SIR epidemic model with negative feedback and nonpharmaceutical intervention has been developed. The SAIRDM model has five subpopulations: susceptible (non isolated), population (S), isolated population (A), infected population (I), recovered population (R), and dead population (D) together with the information index (on aspects of the corresponding disease) variable (M). A detailed discussion about the basic properties and existence–stability has been presented, and moreover the Hopf bifurcation on the endemic equilibrium depending on the information index has been studied. Thus the model shows a more complex and rich dynamics. We consider nonpharmaceutical control to reduce the spread of the disease and the influence of the information index on the susceptible population’s decision to be isolated. This is essential to understand the dynamics of any epidemic disease transmission and to identify the parameters of greater interest, which will help policy-makers in targeting prevention resources for maximum effectiveness.

## The SAIRD model

In this section, we describe the SAIRD model, which is a perturbation of a basic SIR model taking into account isolated population (*A*) and dead population (*D*).

### SAIRD model

The basic SAIRD model is formulated by the following ordinary differential equations:

1 $$\begin{aligned} \textstyle\begin{cases} S'(t) = \alpha N(t)-\beta I(t)\frac{S(t)}{N(t)}+\eta A(t)- (\rho + \alpha ) S(t), \\ A'(t)= \rho S(t)-(\eta +\alpha )A(t), \\ I'(t)= \beta I(t)\frac{S(t)}{N(t)}-(\gamma +\omega +\alpha )I(t), \\ R'(t)= \gamma I(t)-\alpha R(t), \\ D'(t)= \omega I(t), \end{cases}\displaystyle \end{aligned}$$

where $S, A, I,R,D$ are the epidemiological state variables, and $\beta, \alpha, \eta, \gamma, \omega, \rho $ are the parameters that influence population dynamics. Here we introduce into the SIR model the dynamics of deaths from the disease and the nonpharmaceutical intervention.

We normalize the subpopulations as

$$\begin{aligned} s(t)=\frac{S(t)}{N(t)},\qquad a(t)=\frac{A(t)}{N(t)},\qquad i(t)= \frac{I(t)}{N(t)},\qquad r(t)= \frac{R(t)}{N(t)}\quad \text{and}\quad d(t)= \frac{D(t)}{N(t)}. \end{aligned}$$

Then equation (1) is equivalent to the system

2 $$\begin{aligned} \textstyle\begin{cases} S'(t) = \alpha (1-S(t))-\beta I(t)S(t)+\eta A(t)-\rho S(t), \\ A'(t)= \rho S(t)-(\eta +\alpha )A(t), \\ I'(t)= \beta I(t)S(t)-(\gamma +\omega +\alpha )I(t), \\ R'(t)= \gamma I(t)-\alpha R(t), \\ D'(t)= \omega I(t). \end{cases}\displaystyle \end{aligned}$$

## SAIRD model with negative feedback

We add a feedback variable caused by the information to the SAIRD model; this variable called the information index identifies how misinformation can influence nonpharmaceutical (isolation, quarantine, etc.) or pharmaceutical measures (vaccines, etc.) that we take into account for dynamics of epidemics or pandemics caused by communicable diseases.

**Figure 1:**
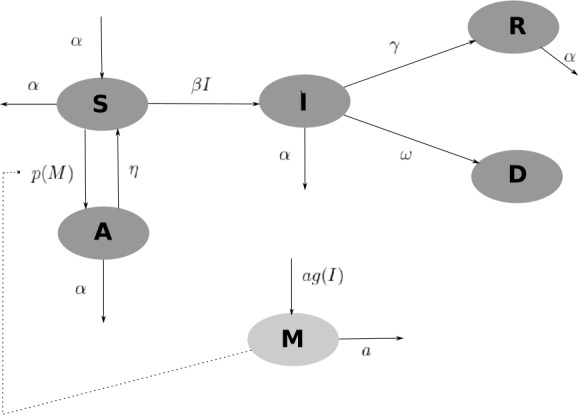
SAIRD model. Diagram of SAIRD model with negative feedback

An epidemic model $SAIRD$ with a period of temporary isolation and negative feedback (see Fig. 1) is given by

3 $$\begin{aligned} \textstyle\begin{cases} S'(t) = \alpha (1-S(t))-\beta I(t)S(t)+\eta A(t)-p(M)S(t), \\ A'(t)= p(M)S(t)-(\eta +\alpha )A(t), \\ I'(t)= \beta I(t)S(t)-(\gamma +\omega +\alpha )I(t), \\ R'(t)= \gamma I(t)-\alpha R(t), \\ D'(t)= \omega I(t), \\ M(t)= \int _{-\infty}^{t} g(S(\tau ),I(\tau ))K_{a}(t-\tau )\,d\tau, \end{cases}\displaystyle \end{aligned}$$

where $S, A, I, R$, and *D* are the population densities of susceptible, isolated, infected, recovered, and dead individuals, respectively, *M* is the information index (on aspects of the corresponding disease), $g(S,I)$ is the influence of the number of susceptible and infected in the information dynamics, $p(M)S$ is the effect of information feedback on the disease isolation rate, *α* is the rate of birth and/or natural death of every individual, *γ* is the recovery rate, *ω* is the death rate due to infection, *τ* is the delay of the effect of the feedback of the information in the isolation policy of the susceptible to avoid the contagion of the disease, and $K_{a}(t-\tau )$ is the selected kernel of the delay. In this work, we use the weak exponential delay kernel $K_{a}(t -\tau ) = ae^{at}$, where *a* is the constant that represents the inverse of the average delay of the information collected about the illness. With this choice, the infinite-dimensional system (3) is equivalent to the following finite-dimensional system of nonlinear ordinary differential equations:

4 $$\begin{aligned} \textstyle\begin{cases} S'(t) = \alpha (1-S(t))-\beta I(t)S(t)+\eta A(t)-p(M)S(t), \\ A'(t)= p(M)S(t)-(\eta +\alpha )A(t), \\ I'(t)= \beta I(t)S(t)-(\gamma +\omega +\alpha )I(t), \\ R'(t)= \gamma I(t)-\alpha R(t), \\ D'(t)= \omega I(t), \\ M'(t)= ag(I)-aM(t). \end{cases}\displaystyle \end{aligned}$$

We consider the following assumptions on the coverage function of the nonpharmaceutical intervention $p(M)$:

- $p(0) > 0$, $p(M) > 0$ for all $M > 0$, and $p^{\prime}(M) > 0$ for all $M > 0$;
- for the function *g*, which characterizes the influence of the infected population on the information, we suppose that $g(0) = 0$, $g(I) > 0$ for all $I > 0$, and $g'(I) > 0$ for all $I > 0$.

### Equilibrium points and basic replacements ratios

The system has a unique disease-free equilibrium (DFE)

$$\begin{aligned} P_{0}=\bigl(S^{*}, 1-S^{*}, 0, 0, 0,0 \bigr), \end{aligned}$$

where $S^{*}=\frac{\eta +\alpha}{\eta +\alpha +p(0)}$.

Now we define the basic reproduction number (epidemic threshold) of the system

$$\begin{aligned} \mathcal{R}_{0} =\frac{\beta}{\gamma +\omega +\alpha} \end{aligned}$$

and the replacement ratio

$$\begin{aligned} \mathcal{R}_{1} =\frac{\eta +\alpha}{\eta +\alpha +p(0)}, \end{aligned}$$

which guarantees that there is always nonpharmaceutical intervention.

#### Theorem 3.1

*The SAIRDM model admits two equilibrium points*:

- *the disease*-*free equilibrium* $P_{0}$, *which always exists*, *and*
- *the endemic equilibrium* $P_{1}$, *which exists if* $\mathcal{R}_{0}\mathcal{R}_{1} > 1$.

#### Proof

The endemic equilibrium point is given by

$$\begin{aligned} P_{1}= \biggl(\frac{1}{\mathcal{R}_{0}}, \frac{p(g(I_{1}))}{(\eta +\alpha )\mathcal{R}_{0}}, I_{1}, \frac{\gamma}{\alpha}I_{1}, D^{*}, g(I_{1}) \biggr), \end{aligned}$$

where $D^{*}=1-(S^{*}+A^{*}+I^{*}+R^{*})$. With $S^{*}=\frac {1}{\mathcal{R}_{0}}$ and $S'= 0$ in the first equation of system (3), we get a solution $I_{1}$ of

$$\begin{aligned} \biggl(1- \frac{1}{\mathcal{R}_{0}} \biggr)- \biggl( \frac{\gamma +\omega +\alpha}{\alpha} \biggr)I_{1}= \frac{p(g(I_{1}))}{(\eta +\alpha )\mathcal{R}_{0}}. \end{aligned}$$

Define

$$\begin{aligned} f_{1}(I)= \biggl(1- \frac{1}{\mathcal{R}_{0}} \biggr)- \biggl( \frac{\gamma +\omega +\alpha}{\alpha} \biggr)I,\qquad f_{2}(I)=\frac{p(g(I))}{(\eta +\alpha )\mathcal{R}_{0}}. \end{aligned}$$

Therefore $I_{1}$ is the solution of $f_{1}(I_{1}) = f_{2}(I_{1})$. Since $f_{1}(I)$ is decreasing and $f_{2}(I)$ is increasing, there is only one solution; moreover,

$$\begin{aligned} f_{1}(0)=1- \frac{1}{\mathcal{R}_{0}}> \frac{p(0)}{(\eta +\alpha )\mathcal{R}_{0}}=f_{2}(0)\quad \text{if } \mathcal{R}_{0}\mathcal{R}_{1}>1, \end{aligned}$$

and

$$\begin{aligned} f_{1}(1)=- \frac{1}{\mathcal{R}_{0}}-\frac{\omega +\alpha}{\alpha}< \frac{p(g(1))}{(\eta +\alpha )\mathcal{R}_{0}}=f_{2}(1). \end{aligned}$$

Then there exists a unique solution $I_{1} \in <0,1> $ if $\mathcal{R}_{0}\mathcal{R}_{1} > 1$. □

## Local and global stability, Hopf bifurcation

In this section, we analyze the local and global stability of the disease-free equilibrium and the global stability of the endemic equilibrium. We can check that the variables *R* and *D* do not appear in the first three equations of the system.

Therefore, we can omit the last two equations of dynamical system (4) and focus on the system

5 $$\begin{aligned} \textstyle\begin{cases} S'(t) = \alpha (1-S)-\beta I(t)S(t)+\eta A(t)-p(M)S(t), \\ A'(t)= p(M)S(t)-(\eta +\alpha )A(t), \\ I'(t)= \beta I(t)S(t)-(\gamma +\omega +\alpha )I(t), \\ M'(t)= ag(I)-aM(t). \end{cases}\displaystyle \end{aligned}$$

### Local stability of the disease-free equilibrium

Let $P_{0}$ be the disease-free equilibrium (DFE),

$$\begin{aligned} P_{0}= \biggl(\frac{\alpha +\eta}{\alpha +\eta +p(0)}, \frac{p(0)}{\alpha +\eta +p(0)}, 0, 0 \biggr). \end{aligned}$$

#### Theorem 4.1

*If* $\mathcal{R}_{0} \mathcal{R}_{1} < 1$, *then the disease*-*free equilibrium* $P_{0}$ *is locally stable*.

#### Proof

Linearizing the system at DFE for these infectious variables and substituting the solution of the form $X(t) = e^{\lambda t}v$, $v \in \mathrm{R}^{4}$, into the matrix equation corresponding to the system, we get the characteristic polynomial

$$\begin{aligned} p(\lambda ) = \det|\lambda I-A|, \end{aligned}$$

where $A = J|_{P_{0}}$. This polynomial has the roots

$$\begin{aligned} &\lambda _{1} = (\omega +\gamma +\alpha ) (\mathcal{R}_{0} \mathcal{R}_{1} - 1), \\ &\lambda _{2}= -a, \end{aligned}$$

and the other two are roots of the quadratic polynomial

$$\begin{aligned} \lambda ^{2} +\theta \lambda +\omega =0, \end{aligned}$$

where $\theta = p(0)+\eta +2\alpha $ and $\omega = \alpha (p(0)+\eta +\alpha )$. The root $\lambda _{1} = (\omega +\gamma +\alpha )(\mathcal{R}_{0}\mathcal{R}_{1}-1) $ is negative if $\mathcal{R}_{0}\mathcal{R}_{1}< 1$, the root $\lambda _{2} = -a < 0$, and the roots of the polynomial $\lambda ^{2} + \theta \lambda +\omega = 0$ are always negative. □

### Local stability and Hopf bifurcation of the endemic equilibrium

We have proved that the endemic equilibrium

$$\begin{aligned} P_{1}= \biggl(\frac{1}{\mathcal{R}_{0}}, \frac{p(g(I_{1}))}{(\alpha +\eta )\mathcal{R}_{0}}, I_{1}, \frac{\gamma}{\alpha}I_{1}, D^{*}, g(I_{1}) \biggr) \end{aligned}$$

exists if $\mathcal{R}_{0} \mathcal{R}_{1} > 1$.

#### Theorem 4.2

*If* $\mathcal{R}_{0} \mathcal{R}_{1} > 1$ *and the coefficients* $B_{i}>0$, $i = 2, 1, 0$ (*independent of the parameter a*), *then the endemic equilibrium* $P_{1}$ *is locally and asymptotically stable*.

#### Proof

The characteristic equation corresponding to this equilibrium point is of the form

$$\begin{aligned} \lambda ^{4} + b_{3}\lambda ^{3} + b_{2} \lambda ^{2} + b_{1}\lambda + b_{0} = 0 \end{aligned}$$

with coefficients

6 $$\begin{aligned} \begin{aligned}& b_{3} = a+\alpha +\Delta >0, \\ &b_{2}= (1+a) (\alpha +\Delta )+\beta I_{1}(\eta +\omega + \gamma + \alpha )>0, \\ &b_{1} = a(\alpha \Delta +\beta \eta I_{1}+\Lambda )+\beta (\eta + \alpha )I_{1}(\omega +\gamma +\alpha )>0, \\ &b_{0}=\alpha \bigl(\beta (\eta +\alpha )I_{1}(\omega + \gamma +\alpha )+ \alpha \Lambda \bigr)>0, \end{aligned} \end{aligned}$$

where $\Delta = \eta +\alpha +\beta I_{1} + p(g(I_{1})) > 0$ and $\Lambda = (\omega +\gamma + \alpha )I_{1} g'(I_{1})p'(g(I_{1})) > 0$. The positivity of the $b_{i}$ rules out the possibility of positive real eigenvalues (Descartes’ theorem). Consequently, the loss of stability can only occur via a Hopf bifurcation.

Since the value of the parameter *a*, which represents the inverse of the average delay of the information collected about the disease, only affects the stability of the endemic point, we will use this *a* as the bifurcation parameter. Define

$$\begin{aligned} &C_{1} = \alpha +\Delta >0, \\ &C_{2}= c_{1}+\beta I_{1}(\eta +\omega +\gamma +\alpha )>0, \\ &C_{3} = \alpha \Delta +\beta \eta I_{1}+\Lambda >0, \\ &C_{4}=\beta (\eta +\alpha )I_{1}(\omega +\gamma +\alpha )>0. \end{aligned}$$

By the Routh–Hurwitz theorem, if $b_{3}b_{2}b_{1}-b_{1}^{2}-b_{0} > 0$, then the endemic point is locally and asymptotically stable. When observing the coefficients $b_{i}$ defined in (6), this last condition is equivalent to the positivity of the cubic polynomial

7 $$\begin{aligned} f(a) = B_{3}a^{3} + B_{2}a^{2} + B_{1}a + B_{0}, \end{aligned}$$

where

$$\begin{aligned} &B_{3} = C_{1}C_{2} >0, \\ &B_{2}= C_{1}C_{4}+C_{1}^{2}C_{2}-C_{2}^{2} -1, \\ &B_{1} = C_{2}C_{4}+C_{1}^{2}C_{4}+C_{1}C_{2}^{2} -2C_{2}C_{4}-2C_{1}b_{0}, \\ &B_{0}=C-1C_{2}C_{4}-C_{4}^{2} -C_{1}^{2} b_{0}. \end{aligned}$$

Only the first coefficient is guaranteed to be positive, and the others are variable. Therefore, if we require that the remaining $B_{i}$, $i = 2, 1, 0$, are positive, then the endemic point is locally and asymptotically stable regardless of the delay, whereas if any of them is negative, then we obtain the possible instability of the endemic point. □

#### Theorem 4.3

*If* $\mathcal{R}_{0} \mathcal{R}_{1} > 1$ *and the coefficients* $B_{i}>0$, $i = 2, 1, 0$, *then there exist two values* $0< a_{1}< a_{2}$ *of the delay parameter* *a* *such that the endemic equilibrium *$P_{1}$ *is unstable for* $a\in (a_{1},a_{2})$, *whereas it is locally asymptotically stable if* $a\in (0.a_{1})$ *or* $a\in (a_{2},\infty )$. *At the values* $a_{1}$ *and* $a_{2}$, *Hopf bifurcations occur*.

#### Proof

As $B_{3}>0$, the cubic polynomial (7) has one negative real root, and if $B_{0}>0$, then the other two roots can be

- two real positive distinct roots, or
- one real positive root of multiplicity 2, or
- two complex roots.

In the last two cases, the endemic point is always locally asymptotically stable independently of *a*. In the first case, since $f(0)>0$, we have $f(\infty )>0$; the endemic equilibrium is always locally asymptotically stable for both small and large values of information delay parameter (this means large or small values of parameter *a*). Then there exist two positive values $a_{1}$ and $a_{2}$ ($a_{1}< a_{2}$) of the delay parameter *a* for the equation $f(a)=0$, which determines two bifurcating values of the delay parameter *a*. In consequence, $f(a)$ is positive for $0< a< a_{1}$ and $a>a_{2}$, which means that the endemic point is locally asymptotically stable, whereas the endemic point is unstable for $a \in [a_{1}, a_{2}]$.

Finally, the following transversality condition for a Hopf bifurcation is satisfied:

$$\begin{aligned} \biggl[\frac {d(b_{3}b_{2}b_{1}-b_{1}^{2}-b_{0})}{da} \biggr]_{a=a_{i}}= \biggl[\frac {df(a)}{da} \biggr]_{a=a_{i}} \neq 0. \end{aligned}$$

 □

### Global stability of the disease-free equilibrium

#### Theorem 4.4

*If* $\mathcal{R}_{0} < 1$, *then the disease*-*free equilibrium is globally and asymptotically stable*.

#### Proof

The disease-free equilibrium $P_{0}$ always exists. In the reduced system, defining $\phi (t) = S(t) + A(t) + I(t)$ with derivative $\phi '(t) = S'(t) + A'(t) + I'(t)$, we get

$$\begin{aligned} \phi '(t)\leq \alpha -\alpha \phi (t). \end{aligned}$$

By a comparison theorem for ODEs (Hale, 1969) we get that $S+A+I \leq 1$. In consequence, if we replace it in the infected population dynamics, then we obtain

$$\begin{aligned} I'\leq I\bigl[\beta -(\omega +\gamma +\alpha )\bigr]. \end{aligned}$$

As

$$\begin{aligned} \mathcal{R}_{0} = \frac{\beta}{\gamma +\omega +\alpha} < 1, \end{aligned}$$

this implies $I(t) \rightarrow 0$ and $S(t) + A(t) \rightarrow 1$. Therefore the disease-free equilibrium is globally and asymptotically stable. □

## Sensitivity analysis

Most of the parameters of the models can have uncertain values; this is known as the uncertainty of the parameters [17, 18]. The best-known technique to detect uncertainty is the Latin hypercube sampling (LHS). On the other hand, for those input parameters, there can be output parameters. In this way the uncertainty analysis can be extended to study the sensitivity analysis, that is, which output parameters are more sensitive to the input parameters. To perform a sensitivity analysis, there are also many techniques; one of them is called the partial rank correlation coefficient (PRCC).

**Figure 2:**
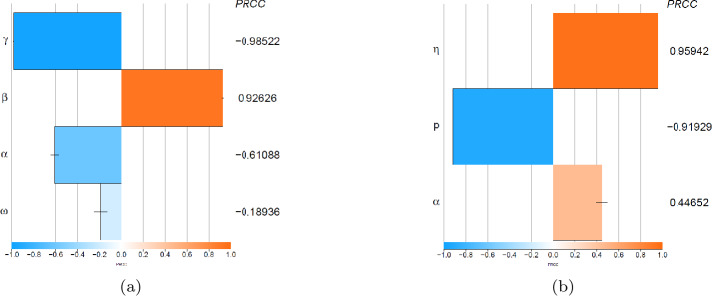
Sensitivity Analysis with (**a**) $\mathcal{R}_{0}$ and (**b**) $\mathcal{R}_{1}$ as the outputs. The input parameters [a] $\beta, \gamma,\omega,\alpha $ and [b] $\eta, \alpha, p(0)$ were chosen from a uniform distribution using Latin hypercube sampling and transforming both class of parameters by partial rank correlation coefficient

We achieved a global analysis of sensitivity of input parameters $\beta, \gamma,\omega, \alpha $ on the variation of the output parameter $\mathcal{R}_{0}$ and of $\eta, \alpha, p(0)$ on the variation of the output $\mathcal{R}_{1}$ in accordance with [17, 18]. First, we get a sample of 5000 input parameters using LHS with $\beta \sim \operatorname{Unif}(0.5, 1)$, $\gamma \sim \operatorname{Unif}(0.1, 0.99)$, $\omega \sim \operatorname{Unif}(0.1, 0.4)$, and $\alpha \sim \operatorname{Unif}(0.0000108, 0.123)$. Figure 2 shows the PRCC obtained from the sensitivity analysis of the output parameter $\mathcal{R}_{0}$. Increasing $\gamma, \alpha $, and *ω* leads to a decrease in $\mathcal{R}_{0}$, and with 93%, increasing the transmission rate *β* promotes an increase in $\mathcal{R}_{0}$. With $\eta \sim \operatorname{Unif}(0.000001, 1)$. Figure 2 shows the PRCC of the output $\mathcal{R}_{1}$. Increasing *p* leads to a decrease in $\mathcal{R}_{1}$, and with 96.1%, increasing the nonpharmaceutical intervention rate *η* promotes an increase in $\mathcal{R}_{1}$. In the same way, increasing the transmission rate *α* promotes an increase of $\mathcal{R}_{1}$ with less percentage.

## Simulations

In this section, we consider some approximations of the parameters according to Peru. Suppose a constant population, the birth and natural death rates are equal, remain constant in time with numerical value equal to the inverse of life expectancy at birth [19]. Here we consider the Peruvian life expectancy of 75 years [20]; in consequence,

$$\begin{aligned} \alpha =\frac {1}{76 \times 365} = 0.000036 \bigl( \text{days}^{-1} \bigr), \end{aligned}$$

and the recovery rate is the inverse of the number of days that the infection lasts. In this case, we will take measles as an example (it has seven days of infectivity), and therefore

$$\begin{aligned} \gamma =\frac {1}{7} = 0.143. \end{aligned}$$

We consider the following assumptions

- the birth, death, isolation, desertion, infection, recovery, and death from infection rates remain constant over time;
- the average number of days of information delay starts from the hypothetical case of immediate information ($\tau = 0$) to a delay of 120 days;
- the parameter values are taken in such a way that they show different scenarios.

For the following simulations, the values of the other parameters were mentioned in Table 1.

**Table 1:** Description, values, and range of parameters of the SAIRDM model

Parameters	Meaning	Value	Range	Reference
*α*	Birth and natural death rate	0.000036	0	Varying
*β*	Transmission rate	0.95	(0.5, 1)	Guessed
*η*	Desertion of a nonpharmaceutical intervention rate	0.00001, 0.3	–	Varying
*γ*	Recovery rate	1/7	(0.1, 0.99)	Estimated
*ω*	Death rate due to infection	0.028	–	Varying
*a*	Inverse of the average delay of the information	0.00833, 0.9	(0.008, ∞)	[13]
*p*	Information-dependent nonpharmaceutical intervention rate	0.75	(0.2, 1)	Guessed
*k*	Information coverage	0.8	(0.2, 1)	[13]

**Figure 3:**
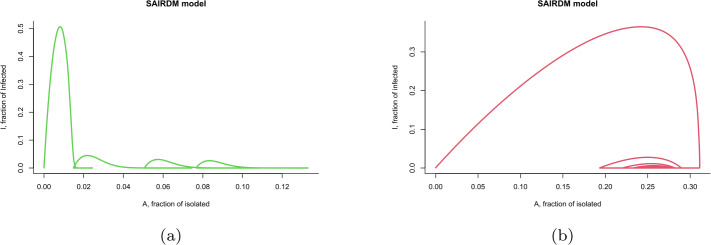
Phase plane: Isolated (*A*)–Infected (*I*) with two different values of the delay parameter *a*: (**a**) $a = 0.00833$, (**b**) $a = 0.9$

For $\eta =0.00001$ (low isolated desertion), the simulations in Fig. 3 correspond to the dynamics of the evolution of a disease considering the nonpharmaceutical intervention and the information index. We consider a total population of susceptible and only one infected. According to the SAIRDM model, we can say that the infected and isolated human population oscillates over time and tends to an equilibrium when the delay parameter *a* is close to one. On the other hand, if the average delay of information is $\tau =120$ d, then the infected population increases when the isolated population is lower, but when the isolated population begins to increase, the infected population tends to decrease and seems to tend to zero as the isolated population decreases. There exists more waves over time, but the peaks are lower than the previous one.

**Figure 4:**
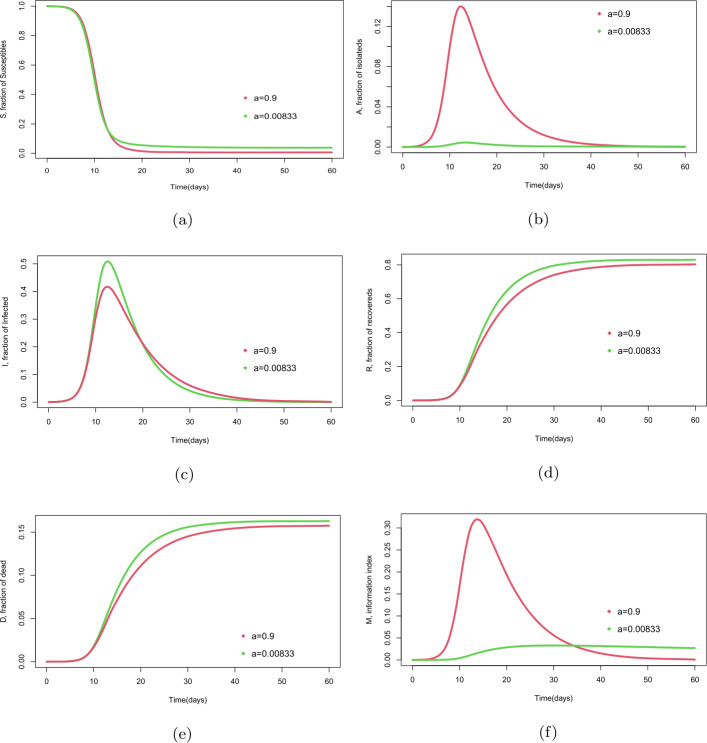
Comparison of evolution of fraction of epidemic populations and information index with two different values of the delay parameter *a*. Populations: (**a**) susceptible, (**b**) isolated, (**c**) infected, (**d**) recovered, (**e**) dead, and (**f**) information index

In Fig. (4), for $\eta =0.3$ (high isolated desertion) and $a=0.9$ regarding the isolated human population that started with zero individuals, we observe that the isolated population starts to increase reaching its maximum value approximately on day 12, with more than 14% of individuals to later descend until it seems to disappear. As the days go by (due to the isolation process and the influence of index of information), the symptoms develop, becoming an infected population, which reaches the highest level approximately on day 12 with 50% of people infected, and then begins to decline until it is close to zero. Finally, the number of recovered individuals increases progressively until day 38, after which it starts to stabilize in approximately 78% of individuals.

The simulations in Fig. 4 of model (4) show the dynamics of evolution of the disease considering two different values of the delay parameter *a*, which shows the negative feedback of information index into the isolation process (nonpharmaceutical intervention).

**Figure 5:**
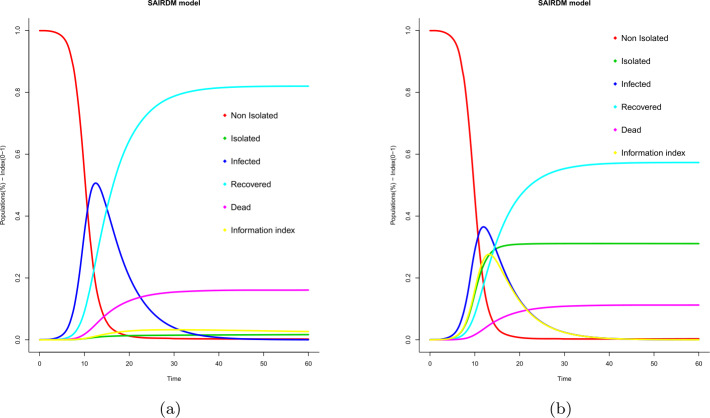
Evolution of fraction of epidemic populations ($S-A-I-R-D$) and information index (*M*). Cases: (**a**) $a = 0.00833$, (**b**) $a = 0.9$

Finally, the simulations in Fig. 5 show the dynamics of evolution of all the epidemiological populations and the information index when the value of the delay parameter varies. We can check that the infected population tends to zero in different scenarios and also that its maximum peak value reaches 50% of the total population in case $a=0.00833$ and 39% in case $a=0.9$. It means that if the information delay parameter *a* has a higher value, then the percentage of infected population is lower (negative feedback).

## Conclusions

In this work, we consider the coupling SAIRD epidemic model with the information index (negative feedback) to investigate the influence of the information in a nonpharmaceutical intervention. The delay, represented by the parameter *a*, identifies the inverse of the time (in days) of delay of the information. We conclude that the fluctuating behavior of the population with respect to the delay in the information affects the nonpharmaceutical intervention and determines the decrease of the incidence rate; in consequence, the health system is prepared to face the epidemic. The SAIRDM model considering the negative feedback of the information identifies the influence of the nonpharmaceutical intervention and desertion strategy in the spread of an infectious disease. There are some parameters in the model that determine the sensibility in the dynamics of the SAIRDM model. Theoretically, we found a Hopf bifurcation for the endemic equilibrium determined by the parameter *a*, which allows us to appreciate the switching on stability and instability of the equilibrium. Finally, the perturbations of the basic SIR mathematical model responded to multidisciplinary research questions.

Our future research would also devise a delay in the isolation rate, and the delay parameter would determine the chaotic nature of the disease propagation.

## Data Availability

Not applicable.
